# Can You Feel My Pain? Neural‐Behavioural Changes in Caregiver–Infant Dyads During Ostracism

**DOI:** 10.1111/desc.70269

**Published:** 2026-08-23

**Authors:** Niloofar Goharbakhsh, Louisa Kulke

**Affiliations:** ^1^ Developmental Psychology with Educational Psychology University of Bremen Bremen Germany

**Keywords:** EEG hyper scanning, ostracism, social exclusion, inter‐brain synchrony, theta power

## Abstract

Behavioural and neural coordination during infant‐caregiver social interaction is crucial for early emotional development and effective emotional regulation abilities later in life. This is particularly relevant to help infants deal with challenging social situations, such as being excluded from a group of others (ostracism). The coupling of neural and behavioural responses to ostracism in infants and caregivers has never been investigated in infants and caregivers simultaneously, nor how early infants perceive ostracism and how caregivers couple their behavioural and neural signals with them. In the current preregistered hyperscanning study, we investigated the effect of ostracism on neural and behavioural responses, inter‐brain synchrony (*N* = 34) and behavioural synchrony in caregiver‐infant dyads (*N* = 41) using a ball‐game. We compared how theta power, inter‐brain synchronization, and affective responses changed during ostracism. 5–7‐month‐old infants and their caregivers both showed increased affective responses. However, only infants, but not their caregivers showed higher theta power during ostracism. Inter‐brain neural synchrony was not generally higher in the exclusion condition compared to the control conditions and highly dependent to specific contexts. These findings indicate a co‐regulation process, suggesting that “more synchrony” is not necessarily better for interaction quality. Instead, effective caregiver–infant interaction may rely on the flexible modulation of synchrony, shifting between more and less synchronization depending on contextual demands. Overall, our study is the first to show that infants are sensitive to cues of ostracism experience from 5 months of age on and provides the first evidence of caregiver–infant behavioural and neural synchrony in response to ostracism. A video abstract of this article can be viewed at https://youtu.be/pXj3yu7MNw0.

## 1 Introduction

### 1.1 Ostracism

Social interaction plays an important role in our daily life. Therefore, social exclusion or ostracism—being ignored and excluded from social groups—can threaten primary needs such as belonging, self‐esteem, and a sense of meaningful existence across the life span (Williams 2007; Williams et al. 2000). This phenomenon commonly occurs across the lifespan from infancy to adulthood and in different contexts (e.g., school, romantic relationships, and workplace) (Abrams et al. 2011; Masclet 2003; Testa et al. 2025; Wölfer and Scheithauer 2013; Zadro and Gonsalkorale 2014). In research, the effects of ostracism have been examined using a ball‐tossing game and its online version (Cyberball paradigm), where two experimenters play a ball game with participants and manipulate inclusionary states of participants by either tossing a ball towards them or ostracizing them from the game (Williams et al. 2000).

**Summary**

- This study demonstrates that infants as young as five months old exhibit neural sensitivity (higher theta power) to ostracism.
- Caregivers showed no changes in theta power in response to ostracism, suggesting effective emotion regulation abilities, possibly in place to downregulate infants’ affective responses.
- Infants and caregivers showed behavioural synchrony during ostracism, but neural synchrony depended strongly on the specific context.
- These findings suggest a co‐regulation process during ostracism, highlighting flexible modulation of synchrony rather than consistently higher synchrony as key for effective interaction.

### 1.2 Behavioural Responses to Ostracism in Infancy

Most research has focused on the effect of ostracism on children, adolescents, and adults. Evidence suggests that infants also experience different forms of ostracism early in life, not only from their caregivers (e.g., through separation) but also in interactions with their peers (Hart and Legerstee 2010). Prendergast (2019) investigated how infants between 7 and 9 months react to seeing others being ostracised: They found that infants gazed differently at animations depicting inclusion compared to exclusion. But how do infants react when they are ostracised themselves? To our knowledge, this has never been investigated in infants under 1 year of age. According to Williams’ Temporal Need‐Threat Model (Williams 2007, 2011), individuals experiencing ostracism first reflexively respond with negative emotions (Step 1). Following this, they evaluate the situation (Step 2) and may show either prosocial behaviours, such as attention‐seeking or antisocial behaviours, such as aggression in the reflective stage. Quadrelli et al. (2023) confirmed that 13–14‐month‐old toddlers display negative emotionality in response to ostracism. They also showed increased visual attention and attention‐seeking behaviours, suggesting that they may attempt to reconnect with other players to compensate for their unmet needs. But how early are infants sensitive to ostracism? In the present study, we adapt Quadrelli et al.’s approach to test whether younger infants exhibit similar responses to ostracism.

### 1.3 Neural Responses to Ostracism in Infancy

Responses to ostracism are evidenced not only at the behavioural level but also at the neural level. Several studies highlighted the important role of the medial frontal cortex in regulating negative affect in response to ostracism (Gunther Moor et al. 2012). Quadrelli et al. (2025) showed that 13‐month‐old infants showed increased Nc (indicator of attention allocation) event‐related potentials (ERPs) to emotional pictures after experience of exclusion, suggesting an effect of ostracism on social information processing and the early emergence of a social monitoring system that may facilitate the detection of opportunities for re‐affiliation or potential social threats. Another important neural indicator of ostracism is theta power, which is increased during ostracism (Tang et al. 2021; Van Noordt et al. 2015a). Theta power appears to reflect distinct cognitive‐affective processes over time, with early increases associated with the monitoring of social expectations and the detection of expectancy violations, and later increases related to the appraisal and regulation of the emotional impact of ostracism (Bekkedal et al. 2011; Cristofori et al. 2013). Theta activity is also related to processing error/conflict monitoring (Trujillo and Allen 2007), self‐regulation (Ertl et al. 2013; Knyazev 2007), stress, and anxiety (Sreekrishnan et al. 2014). Given that many of these processes are involved is ostracism, theta power can serve as a neural indicator of ostracism (Van Noordt et al. 2015b). However, to date, no study has examined whether ostracism modulates theta power in early infancy. As infants are pre‐verbal, they cannot explicitly be asked about their ostracism experience. Therefore, non‐verbal measures such as EEG (Kulke et al. 2016) can provide relevant information in infants. The current study aims to examine how infants process ostracism and to investigate whether theta power is modulated by early ostracism experiences.

### 1.4 Vicarious Ostracism and Neural Synchrony

Ostracism affects not only victims but also observers (Giesen and Echterhoff 2018; Wesselmann et al. 2009). Previous research has shown the mere observation of ostracism induces negative emotions, activation in the same brain areas as victims, and increased heart rate and skin conductance (i.e., signs of stress) (Coyne et al. 2011; Eisenberger and Lieberman 2004). Since infants and caregivers share a special emotional connection (Bowlby 1958), we predict that ostracism may affect both infants and their caregivers. This is particularly relevant, as caregivers play a crucial role in infants’ emotion regulation as an external regulator (Feldman 2012) and contingent maternal responsiveness helps infants regulate their negative emotions (Bozicevic et al. 2025; Lowe et al. 2012). In a co‐regulation process, caregivers help their infants to downregulate strong emotions, such as potential negative feelings during exclusion (Feldman et al. 1999; Somers et al. 2022). Although parent–child behavioural synchrony and its association with children's emotion regulation have been well documented (Feldman 2015), little is known about the neural mechanisms underlying these processes, especially during ostracism. According to Konvalinka and Roepstorff (2012), such a neural synchronization mechanism may not solely arise from behavioural coupling but may also reflect inter‐individual top‐down modulations. Electroencephalography (EEG) Hyper scanning allows us to measure responses of multiple brains simultaneously (Montague 2002; Turk, Vroomen et al. 2022) and therefore can uncover this co‐regulation process by mapping contingencies between parents’ and infants’ neural regulation. For example, Liu et al. (2024) showed that neural synchrony between parents and children is associated with shared positive affect and parental emotional warmth. This synchronous coordination supports interpersonal emotional co‐regulation, which in turn promotes the child's ability to self‐regulate emotions and adapt physiologically to social stress (Ambrose and Menna 2013; McDonald and Perdue 2018). In the current study, we investigate the inter‐brain synchrony during ostracism. As brain synchrony is affected not only by intrinsic neural processes, but also by dyad‐specific factors such as attachment quality and parental bonding (Turk, Endevelt‐Shapira et al. 2022), we also investigate how variations in parental bonding affect the neural synchrony.

### 1.5 Current Study

In the present study, we applied EEG hyperscanning to measure neural synchrony of caregivers and infants in frontal/mid‐frontal regions during the ball‐tossing game and its association with parental bonding. Caregiver‐infant dyads played a ball‐tossing game which consisted of three conditions; the inclusion condition (dyad and experimenters have equal opportunity to toss the ball), the exclusion condition (two experimenters ignore the dyad and toss the ball to each other), and the reinclusion condition (two experimenters again toss the ball to the dyad to reinclude them). Moreover, behavioural responses of caregivers and infants were recorded, including emotional expressions, attention‐seeking behaviours, visual attention, and social referencing. We predicted (https://osf.io/dzh9s/overview) that: (1) Infants and caregivers show higher theta power during the exclusion condition compared to the inclusion condition, respectively; (2) Caregiver–infant dyads exhibit enhanced theta‐band neural synchronization compared to permuted dyads and control conditions; and (3) The higher the caregiver's parental bonding score, the more strongly the synchronization between caregiver‐infant dyads. We furthermore expected that (4) negative emotionality increases and positive emotionality decreases in the exclusion condition compared to the inclusion condition in both caregivers and infants, indicating a sensitivity to ostracism; and (5) There is a positive correlation between infants’ and caregivers’ positive and negative emotionality. Regarding attention‐seeking behaviour, we expected that (6) Infants’ and caregivers’ visual attention and attention‐seeking behaviours differ between the inclusion and exclusion conditions, indicating a sensitivity to ostracism; and (7) There is a positive correlation between Infants’ and caregivers’ visual attention and attention‐seeking. Finally, we expected social referencing to occur, with (8) infants and caregivers showing more social referencing in the exclusion condition compared to the inclusion condition.

## 2 Method

### 2.1 Participants

Forty‐five healthy infants (*M* = 6.47 months, SD = 0.89, min = 5.07, max = 7.97, 17 females, 28 males) and their caregivers (*M* = 34.2 years, SD = 3.31, 39, min = 27.72, max = 42.74, 39 females, six males) participated in the current study. We selected 5–7‐month‐old infants to investigate early behavioural and neural responses to ostracism before more advanced social‐cognitive abilities emerge. This age range is based on evidence that by 7–9 months infants can already respond to ostracism in others (Prendergast 2019). Moreover, infants by 6 months show flexible attentional allocation between social and non‐social stimuli (Bremner and Wachs 2010; Fogel 2015), suggesting the presence of relevant precursors in early infancy. They were recruited via a local participant data base and advertisements. Two dyads were excluded due to discomfort with the EEG cap and disinterest in the ball game and due to the experimenter deviating from neutral facial expressions during the ball game; therefore 41 participants were included in the affective and behavioural analyses and the correlation analysis between infants’ and caregivers’ affective and behavioural responses. Additional dyads to the four excluded in behavioural analysis were excluded depending on the type of neural analysis. For theta and alpha power in infants, four infants were excluded due to technical problems (e.g., technical errors in the EEG system and unsaved markers in EEG data), and three infants due to less than 50% of trials remaining for each event, resulting in 34 infants. For theta and alpha power in caregivers, four caregivers were excluded after EEG preprocessing due to the technical problems (e.g., technical errors in the EEG system and unsaved markers in EEG data), resulting in 37 caregiver datasets. For inter‐brain synchrony analysis and correlation between parental bonding and inter‐brain synchrony values, seven dyads were excluded because one or both pairs had less than 50% of valid trials after preprocessing, as well as technical errors in the EEG system and unsaved markers in EEG data, resulting in 34 infant‐caregiver dyads. Fourteen additional dyads were tested to reach the sample size of 34 participants in line with the power analysis for theta power (Supplementary Materials 1). The study was approved by the local ethics committee of the Bremen University (reference number 2024–2011) and conducted in line with the Declaration of Helsinki. Methods, hypotheses, and analyses were preregistered with the Open Science Framework (https://osf.io/c3n6g).

### 2.2 Procedure

**FIGURE 1:**
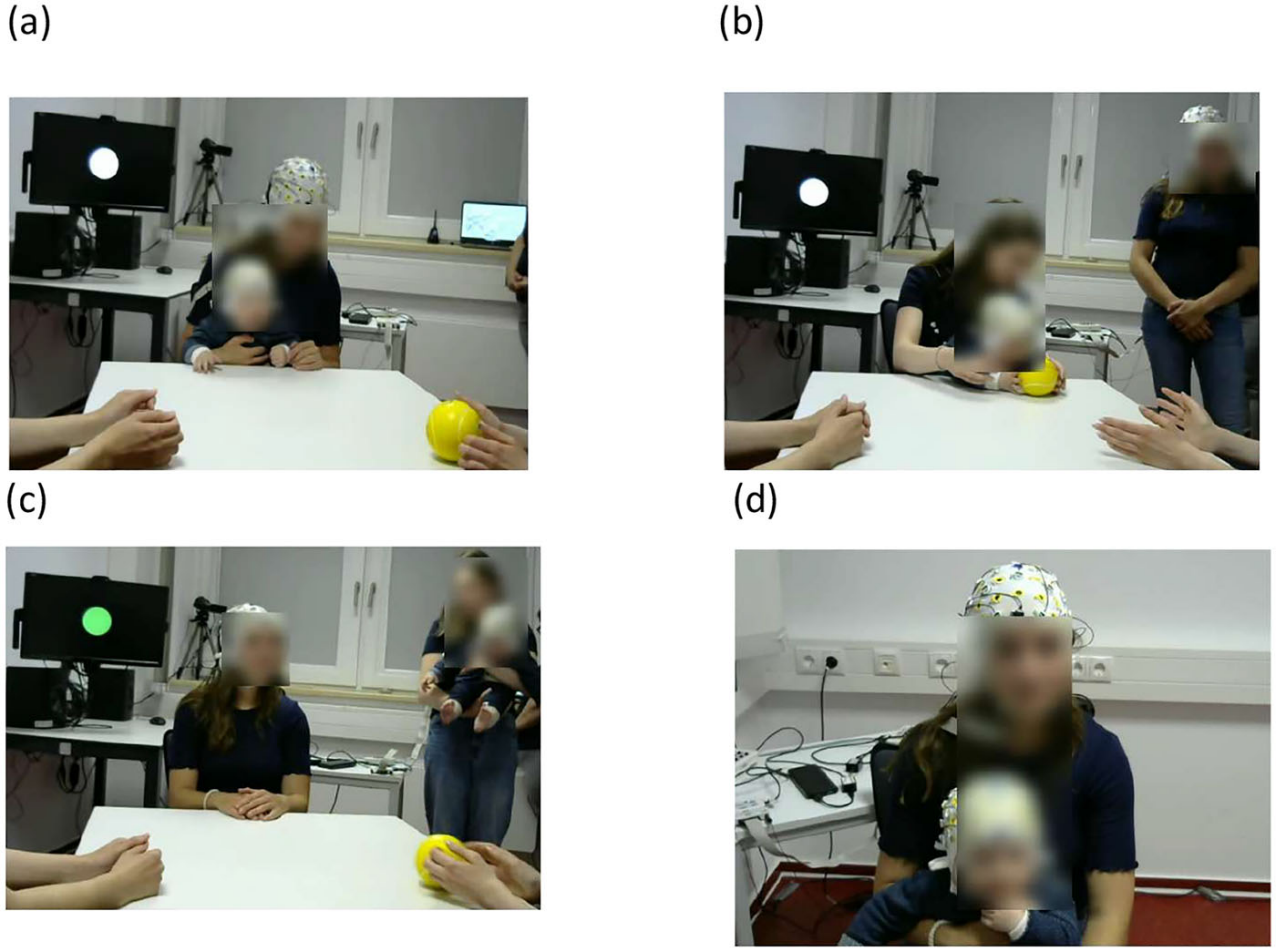
Procedure of the main condition in which the caregiver (wearing the EEG net) plays with the infant (a), infant‐control condition in which an experimenter plays with the infant while the caregiver watches (b), cargiver‐control condition in which the caregiver plays while the infant watches (c) and video‐control condition in which the caregiver and infant watch a video together (d).

Upon arrival, the caregivers read and signed the data protection and informed consent form. After applying EEG for both caregivers and infants, they were invited to an adjacent room to play a ball‐tossing game on the table with two other experimenters. Infants sat on the caregiver's laps. The caregiver supported the infant to toss a ball back and forth with the experimenter. In the inclusion block, all the players had equal opportunity to toss the ball toward each other (14 tosses each). Subsequently, the two experimenters ignored the infant‐caregiver‐dyad and tossed the ball toward each other with equal opportunity (eight tosses each) in the exclusion block. Finally, the experimenters included the infant‐parent‐dyad in the game again in the reinclusion block by tossing the ball towards them (five tosses each). The experimenters maintained a neutral, friendly facial expression and eye contact during the entire game (Figure 1). The game was recorded via the webcam for the behavioural analysis. In order to synchronize the recorded videos with the EEG data, one of the experimenters pressed a button on a small keypad connected via Python/Psychopy, which displayed a green circle on the monitor behind the dyads (outside their visual field) at the start of the ball game and sent markers to the EEG data. In addition, a white circle was displayed every second on the same screen while a simultaneous marker was sent. This procedure enabled us to synchronize the video and EEG recordings and provided accurate markers for subsequent EEG analysis.

Dyads participated in three counterbalanced control conditions if they were willing to continue. In the caregiver condition, the caregiver played the ball game (inclusion and exclusion) with two experimenters while the infant, held by another experimenter within 60 cm, observing the game. In the infant condition, the infant played the game on the experimenter's lap while the caregiver observed from 60 cm away. In the video condition, dyads watched a pre‐recorded ball game (recorded from their own perspective) (Figure 1). After completing the control conditions, caregivers filled out the Postpartum Bonding Questionnaire (PBQ; Reck et al. 2006), demographic information, and were fully debriefed. Finally, infants received a certificate and a small gift for their participation.

### 2.3 Measurement

#### 2.3.1 Affective and Behavioural Responses

During the ball game, behavioural responses were recorded with a Logitech C920 PRO HD camera. The recording started when the experimenter pressed a button on the small keypad, which simultaneously showed the circle on the screen to send markers via a parallel port for EEG synchronization. The video recording and circles on the monitor were controlled via Python/Psychopy.

#### 2.3.2 EEG Recording

EEG was recorded at a sampling rate of 500 Hz from 32 actiCAP slim active electrodes, mounted in the electrode cap (actiCAP snap; Brain Products GmbH, Gilching, Germany). FCz was used as a reference during the online recording, which was filled with gel and kept below 40 kΩ to ensure adequate signal quality. The EEG was recorded on two separate computers (one for the infant and one for the caregiver) using BrainVision Recorder software (v1.21.0402, Brain Products 15 GmbH, Gilching, Germany) for infants and caregivers separately. Electrodes F7, F3, Fz, F4, F8, FC1, FC2, Cz (approximately corresponding to electrode clusters used in a study by Van Noordt et al. (2015a) with different electrode montages) and Oz were filled with electrode gel to maximize efficiency of the set up, and if the infant was happy, additional occipital and parietal electrodes (O1, O2, P3, Pz, P4) were filled with gel for both the infant and caregiver. Electrode impedances were kept below 40 kΩ for all electrodes. For infant participants, electrodes were filled with an infant‐friendly electrode cream.

#### 2.3.3 Postpartum Bonding Questionnaire‐16 (PBQ‐16)

The PBQ was designed as a self‐report scale to identify bonding difficulties (Brockington et al. 2001). In the current study, we used the short version of the PBQ (Reck et al. 2006). Caregivers rated each statement such as, “I feel close to my baby” or “My baby irritates me” on a 6‐point Likert scale ranging from 0 (“never”) to 5 (“always”). Higher PBQ‐16 scores reflect a poor level of bonding. Internal consistency was acceptable (Cronbach's *α* = 0.77), with sum scores ranging from 3 to 27 (*M* = 10.17, SD = 5.52).

### 2.4 Analysis

#### 2.4.1 Affective and Behavioural Coding

Affective and behavioural responses were coded on five scales for infants and caregivers as follows: (1) positive emotionality, (2) negative emotionality, (3) visual attention, (4) attention‐seeking behaviour, and (5) social referencing (See Supplementary Materials 2). The videos were coded by two experimenters and one blind coder and the inter‐rater reliability (Fleiss' Kappa) was computed for around 10% of the videos. Each subscale was coded frame by frame using BORIS software (Friard and Gamba 2016) (See Supplementary Materials 2 for checking the reliability). The scores of the different subscales were normalized by dividing by the total frame subscales of the relevant block and then multiplied by 100 to get a percentage of the sum score. Finally, normalized subscales were summed up to calculate a score for each scale.

#### 2.4.2 EEG Processing

Prior to preprocessing, the video recordings of the ball‐tossing game were coded to generate three types of markers: (1) “inclusion” markers identified at the time when the experimenters tossed the ball toward the infant‐caregiver dyad, (2) “not my turn markers” identified when the experimenters tossed the ball to each other during the inclusion block and (3) “exclusion” markers identified when the experimenters tossed the ball to each other during the exclusion block. The main focus of this study was to compare “not my turn” to “exclusion” conditions as the perceptual characteristics of these events were identical and there was no movement required from the participants in both of these events. All markers were calculated manually based on the green circle indicating the start of the ball game, and the updated markers were implemented on the continuous data of each participant using MATLAB. Moreover, the quality of the reference channel (Oz) was checked before including data in the analyses, based on both the impedance (mean_infant_ = 8.23, SD_infant_ = 6.08, max_infant_ = 28, mean_caregiver_ = 13.09, SD_caregiver_ = 9.49, max _caregiver_ = 39) and visual inspection. EEG data were processed using identical analysis protocols for adult and infant data using the Maryland analysis of developmental EEG (MADE) pipeline (Debnath et al. 2020) in MATLAB using the EEGLAB toolbox (Delorme and Makeig 2004). First, FIR filters with a high‐pass boundary of 2 and a low‐pass boundary of 15 Hz were applied by using FIRfilt plug‐in of EEGLAB (Widmann 2015). This filter was chosen to include the theta and alpha bands of interest in both infants and caregivers, while minimizing slow drifts, movement‐related artifacts, and high‐frequency noise. The wider lower and upper bounds were used to provide a buffer, thereby minimizing edge artifacts. Bad channels were detected and removed by the EEGLAB plug‐in FASTER (Nolan et al. 2010). To ensure the data quality, channels flagged by the FASTER algorithm were also visually inspected to confirm poor signal quality, and the remaining channels were visually checked to ensure they had adequate signal quality. Finally, a copy/ICA procedure was performed on a range of 9 to 14 channels (see Debnath et al. (2020) for details). For this purpose, a copy of the original data was created, a high‐pass filter (1 Hz) was applied to the copy, and channels and epochs with excessive artifacts were removed from the copy. Independent component analysis (ICA) was performed on the copy of the remaining channels of interest, as the rest of the channels were not filled with gel, and then, ICA weights were transferred back to the original data. Artifactual Independent Components (ICs) caused by eye movement were removed from the original data manually based on the visual inspection (component topographies, time courses, and power spectra). The data was epoched around the triggers for inclusion, not my turn and exclusion markers, including one second of data recordings for each condition. A baseline correction was applied using a 200 ms time interval before the trigger. A voltage threshold rejection (±150 µV for infants and ±100 µV for caregivers) was applied to the epochs, and channels were removed if they exceeded the voltage threshold. In the next step, the channels identified by the FASTER function were interpolated. Since it was not possible to fill all electrodes with gel for infants due to time constraints, the data was not re‐referenced to the average. As the aim was to measure theta and alpha power in frontal/midfrontal regions, the data was re‐referenced to the Oz channel. After preprocessing, dyads with fewer than 50% of trials in each condition (Inclusion ≤ 7, Not My Turn ≤ 7, Exclusion ≤ 8) were excluded from further analysis. The overall mean and SD of the number of trials included in all neural analyses are as follows: infants: M_inclusion_ = 12.5, SD_inclusion_ = 1.78, M_exclusion_ = 13.5, SD_exclusion_ = 2.50 and M_not my turn_ = 12.5, SD_not my turn_ = 1.67, caregivers: M_inclusion_ = 12.8, SD_inclusion_ = 1.62, M_exclusion_ = 14.0, SD_exclusion_ = 2.30 and M_not my turn_ = 12.8, SD_not my turn_ = 1.57). (Detailed means and SD for each individual analysis are provided in Supplementary Materials 3 and 4).

#### 2.4.3 Alpha and Theta Power

Alpha and theta power were calculated using the Matlab function pwelch. Each epoch was divided into 50% overlapping 1‐sec segments. Each segment was windowed with a 1‐s Hanning window, and a Fast Fourier Transformation was applied to obtain spectral density. The frequency bands were adjusted to age‐related shifts in frequency band (Cuevas and Bell 2022; Turk, Endevelt‐Shapira et al. 2022): We used frequency bands ranging from 3 to 6 Hz for theta and 6 to 9 Hz for alpha in infants (Orekhova et al. 2006). For adults, we used frequency bands ranging from 4 to 8 Hz for theta power and 8 to 13 Hz for alpha power (Kulke et al. 2023; Strijkstra et al. 2003). The power extractions for each frequency band were then averaged across participants. The mean and SD of the remaining trials after preprocessing for theta and alpha power analyses are provided in Supplementary Materials 3 and detailed alpha power analyses are reported in Supplementary Material 6.

### 2.5 Inter‐brain Synchrony

To calculate caregiver–infant inter‐brain synchrony, Phase‐locking value (PLV) was computed: the EEG data were first filtered to the desired frequency band using a finite impulse response filter (infant: 2.9–6.1 Hz and caregiver: 3.9–8.1 Hz) (Lachaux et al. 1999). After that, the instantaneous phase was extracted using the Hilbert transform. For each trial, the PLV was computed by calculating the complex phase difference between infants’ and caregivers’ signals for each trial (1 s window) (the equation can be found in Lachaux et al. 1999; Marriott Haresign et al. 2023). The PLV was calculated for each of the matched trials between infant‐caregiver dyad and subsequently separated according to each condition (inclusion, exclusion, and no my turn). To assess whether the observed PLV exceeded chance levels, a permuted analysis was performed by pairing infants and caregivers randomly 1000 times and calculating the PLV for permuted dyads in the same vein as for the real dyad to create a null distribution of synchrony values expected by chance. In the next step, we compared the real PLV with the permuted PLV distribution for each condition. Differences between the real and permuted data were assessed using a non‐parametric permutation‐based approach (Cohen 2014; Maris and Oostenveld 2007). The p‐value was calculated as the proportion of permuted values that were equal to or greater than the real PLV. Real PLV values exceeding 95% of the surrogate distribution (p < .05) were considered statistically significant. Subsequently, multiple comparisons across channel pairs were controlled using False Discovery Rate (FDR) correction. Moreover, the observed PLV was compared to the PLV of dyads in the control conditions (infant, caregiver, and video condition) using paired‐t tests. Additional measures of inter‐brain synchrony (power correlation and partial directed coherence (PDC)) are described in Supplementary Materials 5.

## 3 Results

For directional hypotheses, one‐sided tests were conducted; for non‐directional hypotheses, two‐sided tests were carried out (*p* < 0.05).

### 3.1 Affective and Behavioural Outcomes

The final sample size for the affective and behavioural analyses was 41 for both infants and caregivers, separately. The results of the inclusion and exclusion blocks are reported below for infants and caregivers, respectively. Exploratory analyses of the reinclusion block are reported in the Supplementary Materials 10.

### 3.2 Infants

**FIGURE 2:**
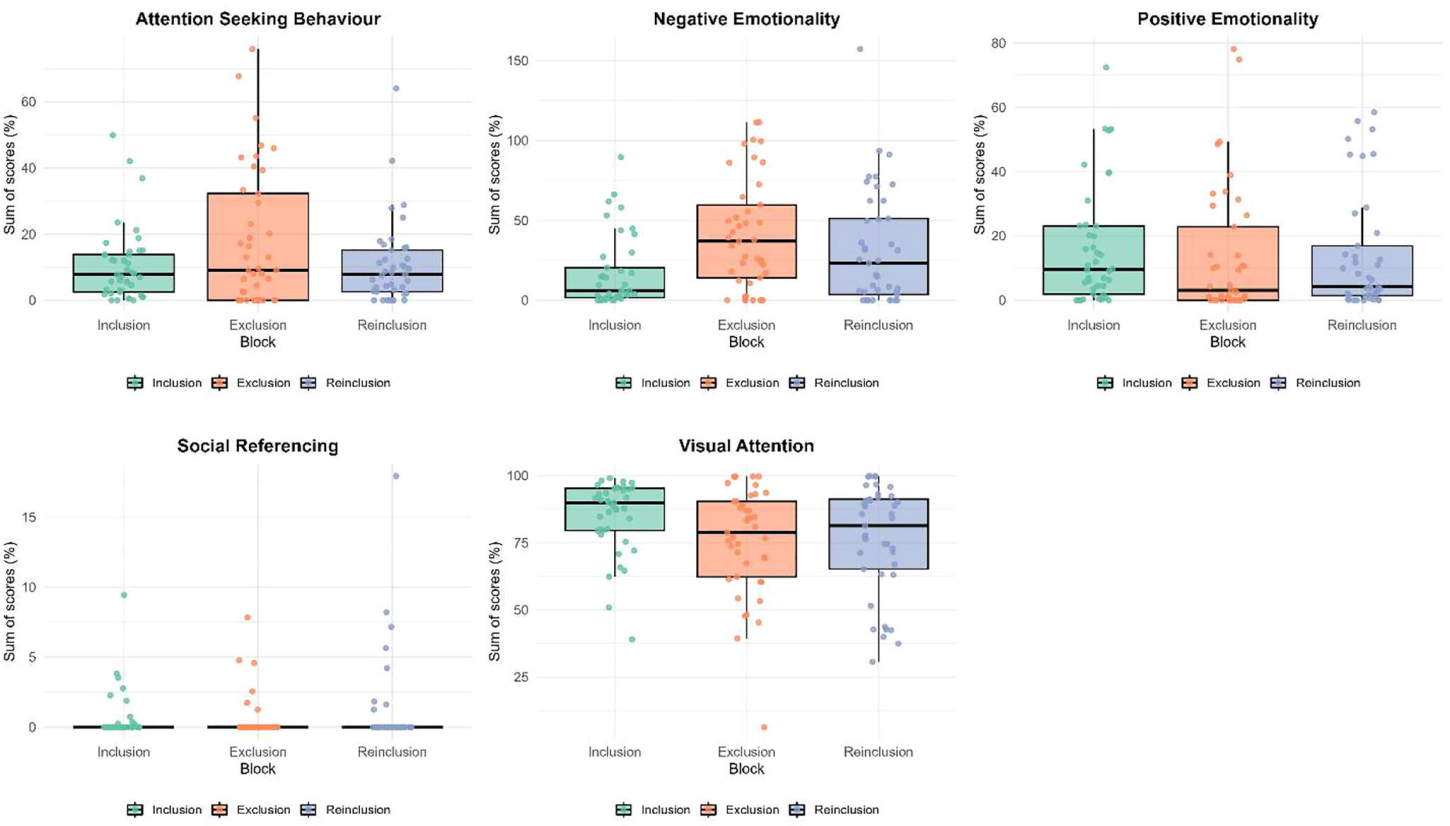
Figures show the percentage of sum scores of attention‐seeking behaviour, negative emotionality, positive emotionality, social referencing and visual attention in infants in inclusion, exclusion and reinclusion blocks.

To test our hypotheses that infants show less positive emotionality and more negative emotionality and social referencing in exclusion compared to inclusion conditions, two paired samples t‐tests and one Wilcoxon test were performed. In addition, Bayes Factors were computed. The result confirmed no significant difference in positive emotionality (M_inclusion_ = 16.01, SD_inclusion_ = 18.18, M_exclusion_ = 13.82, SD_exclusion_ = 20.27), (t (40) = 0.73, p = 0.233, d = 0.11, BF = 3.16) and in social referencing (M_inclusion_ = 0.62, SD_inclusion_ = 1.72, M_exclusion_ = 0.55, SD_exclusion_ = 1.61), (W = 52, p = 1, r = 0.06, BF = 0.17) between inclusion and exclusion conditions. However, infants showed more negative emotionality in exclusion compared to inclusion conditions (M_inclusion_ = 17.03, SD_inclusion_ = 22.41, M_exclusion_ = 41.37, SD_exclusion_ = 34.38), (t (40) = ‐4.62, p < 0.001, d = ‐0.72, BF = 32296.27). To test the hypotheses that there is a difference in attention‐seeking behaviour and visual attention between inclusion and exclusion conditions, we ran one paired samples t‐test and one Wilcoxon test. The result showed no significant difference in attention‐seeking behaviours (M_inclusion_ = 10.55, SD_inclusion_ = 11.24, M_exclusion_ = 18.07, SD_exclusion_ = 20.59), (W = 299, p = 0.137, r = 0.22, BF = 1.40), however, Bayes Factors showed anecdotal evidence for the alternative hypothesis. In terms of visual attention, the result revealed that infants showed more visual attention (M_inclusion_ = 85.09, SD_inclusion_ = 13.45, M_exclusion_ = 75.49, SD_exclusion_ = 20.02), (t (40) = 3.18, p = 0.002, d = 0.50, BF = 12.26) in inclusion compared to exclusion conditions. The behavioural responses of infants are displayed in Figure 2.

### 3.3 Caregivers

**FIGURE 3:**
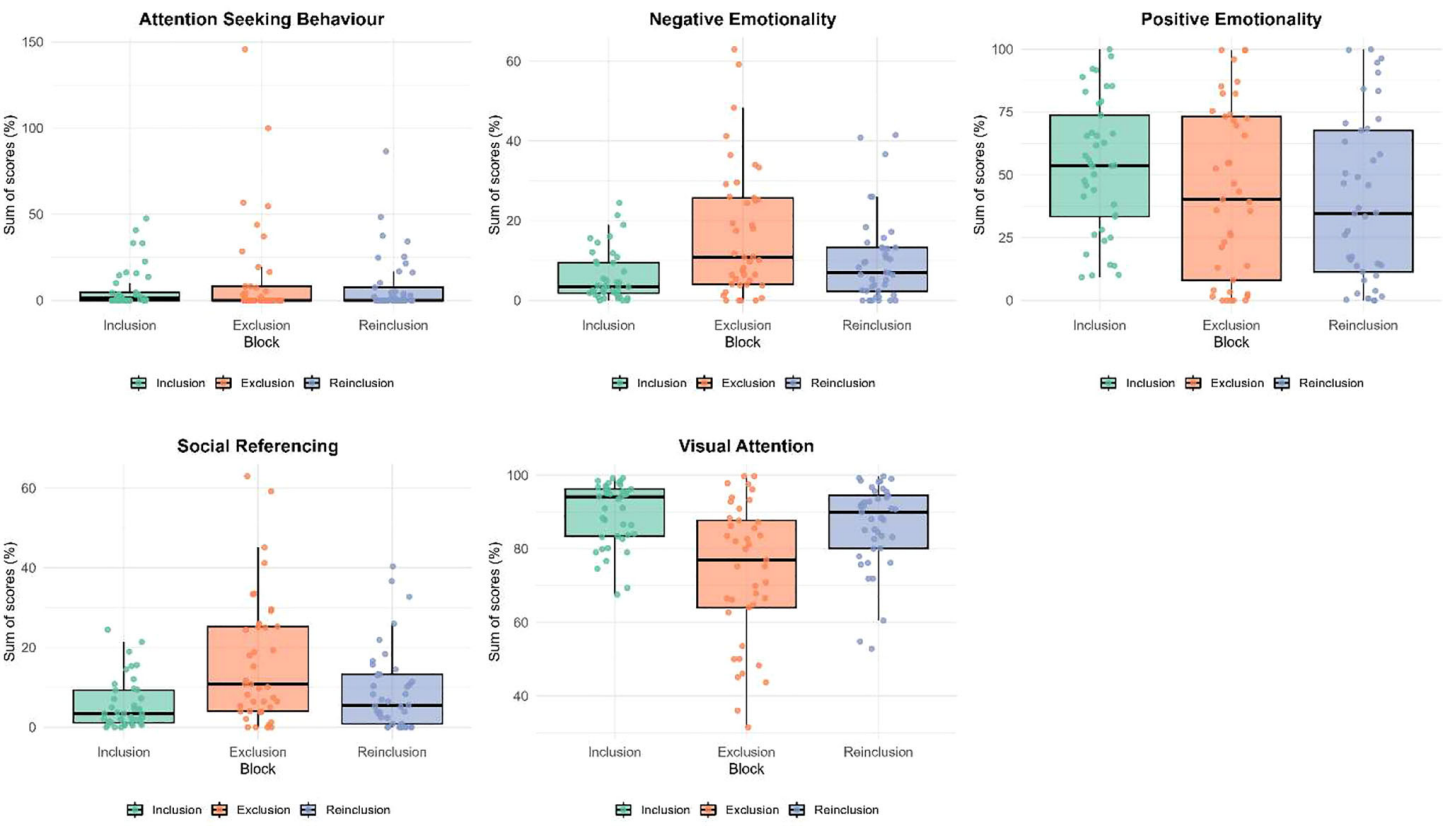
The percentage of sum scores of attention‐seeking behaviour, negative emotionality, positive emotionality, social referencing and visual attention in caregivers in inclusion, exclusion and reinclusion blocks.

To assess whether caregivers exhibit less positive emotionality and more negative emotionality and social referencing in the exclusion compared to the inclusion conditions, one paired samples *t*‐test and two Wilcoxon tests were conducted. In addition, Bayes Factors were computed. Results showed that caregivers showed more positive emotion (M_inclusion_ = 53.3, SD_inclusion_ = 26.55, M_exclusion_ = 43.46, SD_exclusion_ = 34.42), (*t* (40) = 2.32, *p* = 0.012, *d* = 0.36, BF = 66.12) in the inclusion compared to the exclusion condition. Moreover, they showed more negative emotionality (M_inclusion_ = 5.99, SD_inclusion_ = 6.33, M_exclusion_ = 16.9, SD_exclusion_ = 16.29), (W = 78, p < 0.001, r = 0.69, BF = 112529) and social referencing (M_inclusion_ = 5.71, SD_inclusion_ = 6.32, M_exclusion_ = 16.48, SD_exclusion_ = 15.88), (W = 69, *p* < 0.001, *r* = 0.71, BF = 2567.68) in the exclusion compared to the inclusion conditions. To test whether there was a difference in attention‐seeking behaviours and visual attention in the inclusion and exclusion conditions, we ran two Wilcoxon tests. There was no significant difference in attention‐seeking behaviours (M_inclusion_ = 6.95, SD_inclusion_ = 12.05, M_exclusion_ = 13.27, SD_exclusion_ = 29.69), (W = 126, p = 0.213, r = 0.10, BF = 0.92) between exclusion and inclusion conditions. However, there was more visual attention (M_inclusion_ = 89.63, SD_inclusion_ = 8.63, M_exclusion_ = 73.68, SD_exclusion_ = 18.82), (W = 811, *p* < 0.001, *r* = 0.77, BF = 68591.15) in the inclusion compared to exclusion conditions. The behavioural responses of caregivers are displayed in Figure 3.

### 3.4 Correlation Analyses

The final sample size for the correlation analyses between infants’ and caregivers’ affective and behavioural responses was 41 infant‐caregiver dyads.

To investigate the correlation between caregiver and infant on positive emotionality, negative emotionality, attention‐seeking behaviour and social referencing, Spearman correlations were computed as the normal distribution was violated. Correlation analyses showed significant correlations of the positive emotionality (r_s_(39) = 0.26, *p* = 0.048) and negative emotionality (r_s_(39) = 0.33, *p* = 0.015) between caregivers and infants in the inclusion condition. However, there was no significant correlation in attention‐seeking behaviour (r_s_(39) = 0.14, *p* = 0.177) and visual attention (r_s_(39) = 0.08, *p* = 0.292) between caregivers and infants in the inclusion condition. In the exclusion condition, negative emotionality (r_s_(39) = 0.34, *p* = 0.013) and visual attention (r_s_(39) = 0.30, *p* = 0.027) significantly correlated. However, positive emotionality (r_s_(39) = ‐0.00, *p* = 0.516) and attention‐seeking behaviour (r_s_(39) = 0.13, *p* = 0.195) did not significantly correlate in the exclusion condition (Supplementary Materials 11). Regarding social referencing, as most of the infants did not show social referencing, we did not run a correlation analysis for social referencing. Moreover, the results of the correlation analyses for the reinclusion block are provided in Supplementary Materials 11.

### 3.5 Theta Power

#### 3.5.1 Infants

Thirty‐four infants were included in the theta power analysis. The mean and SD of included trials for infants’ analysis and removed ICA components and interpolated channels can be found in Supplementary Materials 3.

**FIGURE 4:**
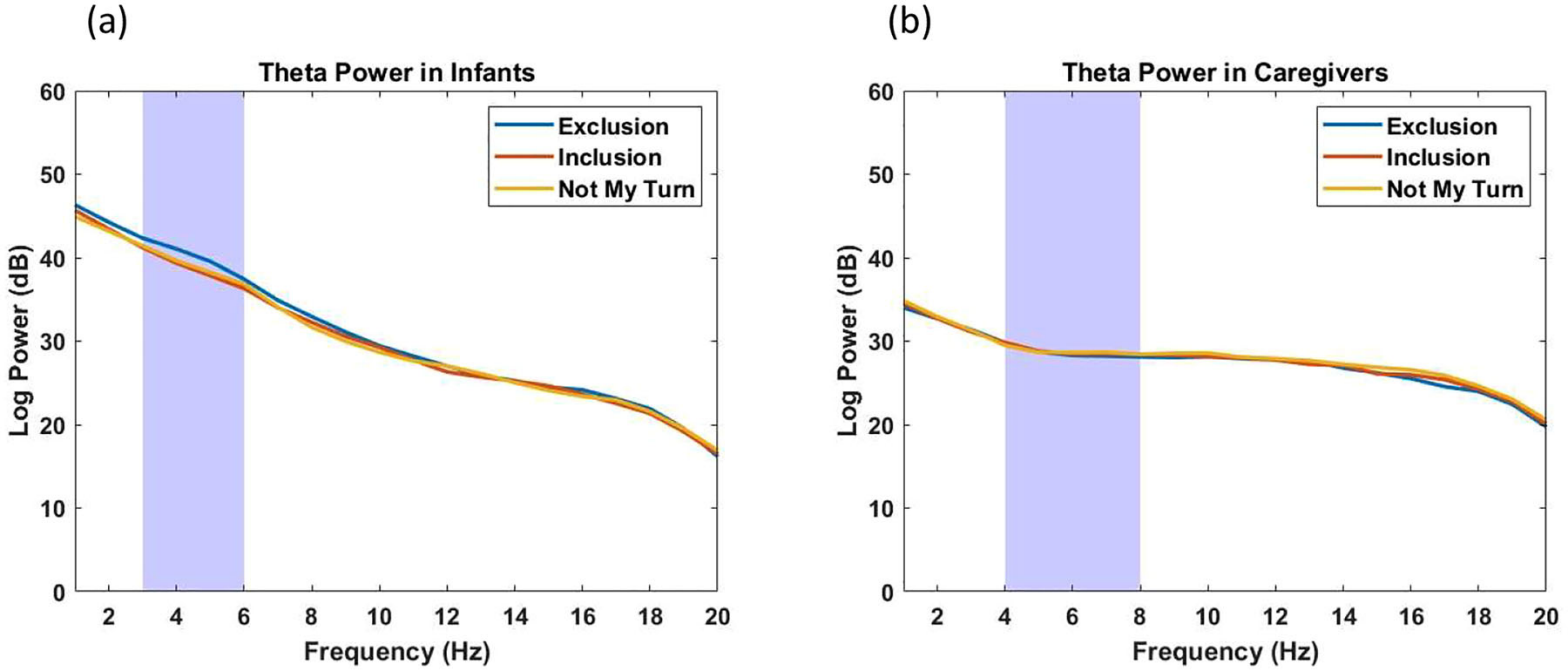
The theta power (purple background) in infants (a) and caregivers (b) during inclusion, exclusion and not my turn trials.

As expected, paired‐samples t‐tests showed a higher theta power during the “exclusion” compared to the “not my turn” events in infants (M_not my turn_ = 8.44, SD_not my turn_ = 0.98, M_exclusion_ = 8.94, SD_exclusion_ = 1.00), (t (33) = ‐2.16, p = 0.018, d = 0.37, BF = 43.76). There was no significant difference in theta power during “exclusion” events compared to “inclusion” events (M_inclusion_ = 8.63, SD_inclusion_ = 1.00, M_exclusion_ = 8.94, SD_exclusion_ = 1.00), (t (33) = ‐1.44, p = 0.078, d = 0.24, BF = 10.65). However, the effect size for the inclusion‐to‐exclusion comparison was smaller than for the exclusion‐to‐not my turn comparison, possibly due to movement confounds (Figure 4).

#### 3.5.2 Caregivers

Thirty‐seven caregivers were included in the theta power analysis The mean and SD of included trials for caregivers’ analysis and removed ICA components and interpolated channels can be found in Supplementary Materials 3.

Theta power was analysed using paired samples t‐tests. The results revealed no difference between the “not my turn” (M_not my turn_ = 5.99, SD_not my turn_ = 1.04, M_exclusion_ = 5.90, SD_exclusion_ = 0.84), (t (36) = 0.46, p = 0.678, d = ‐0.07, BF = 0.48) and “inclusion” events (M_inclusion_ = 5.96, SD_inclusion_ = 0.72, M_exclusion_ = 5.90, SD_exclusion_ = 0.84), (t (36) = 0.40, p = 0.657, d = ‐0.06, BF = 0.53) compared to the “exclusion” events in caregivers (Figure 4).

### 3.6 Inter‐brain Synchrony

A total of 34 infant–caregiver dyads were included in all inter‐brain synchronisation analyses. The mean and SD of included trials and removed ICA components and interpolated channels for all inter‐brain synchrony analyses are provided in Supplementary Material 4.

#### 3.6.1 Phase‐locking Value (PLV)

**FIGURE 5:**
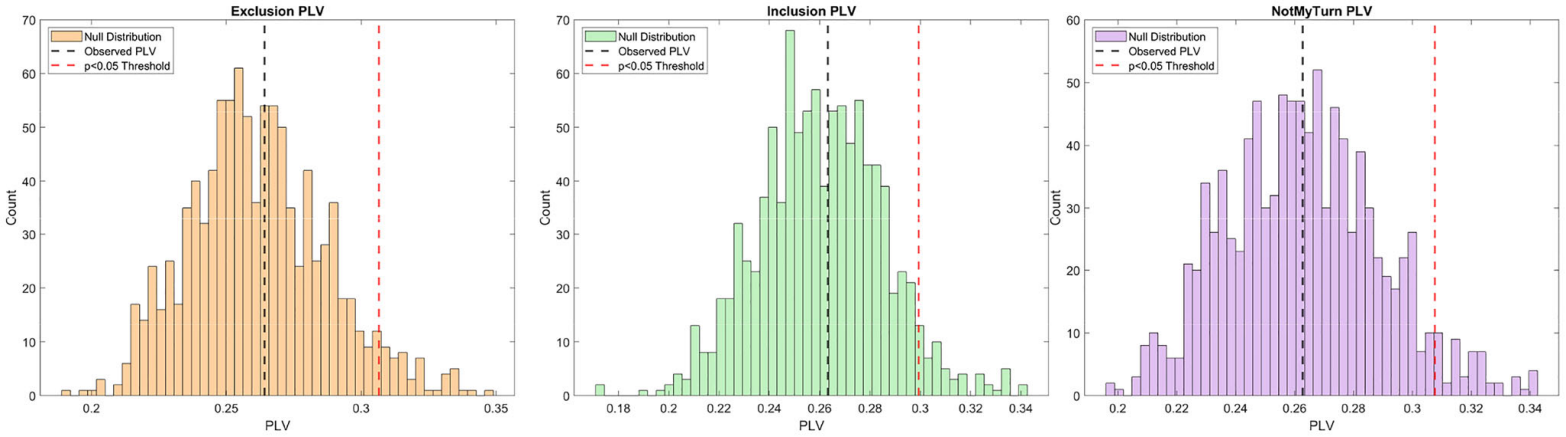
Results of permutation tests for inter‐brain synchrony (PLV) analysis. Red dashed lines indicate averaged observed PLV in Theta, black dashed lines indicate the threshold for *p* < 0.05.

**FIGURE 6:**
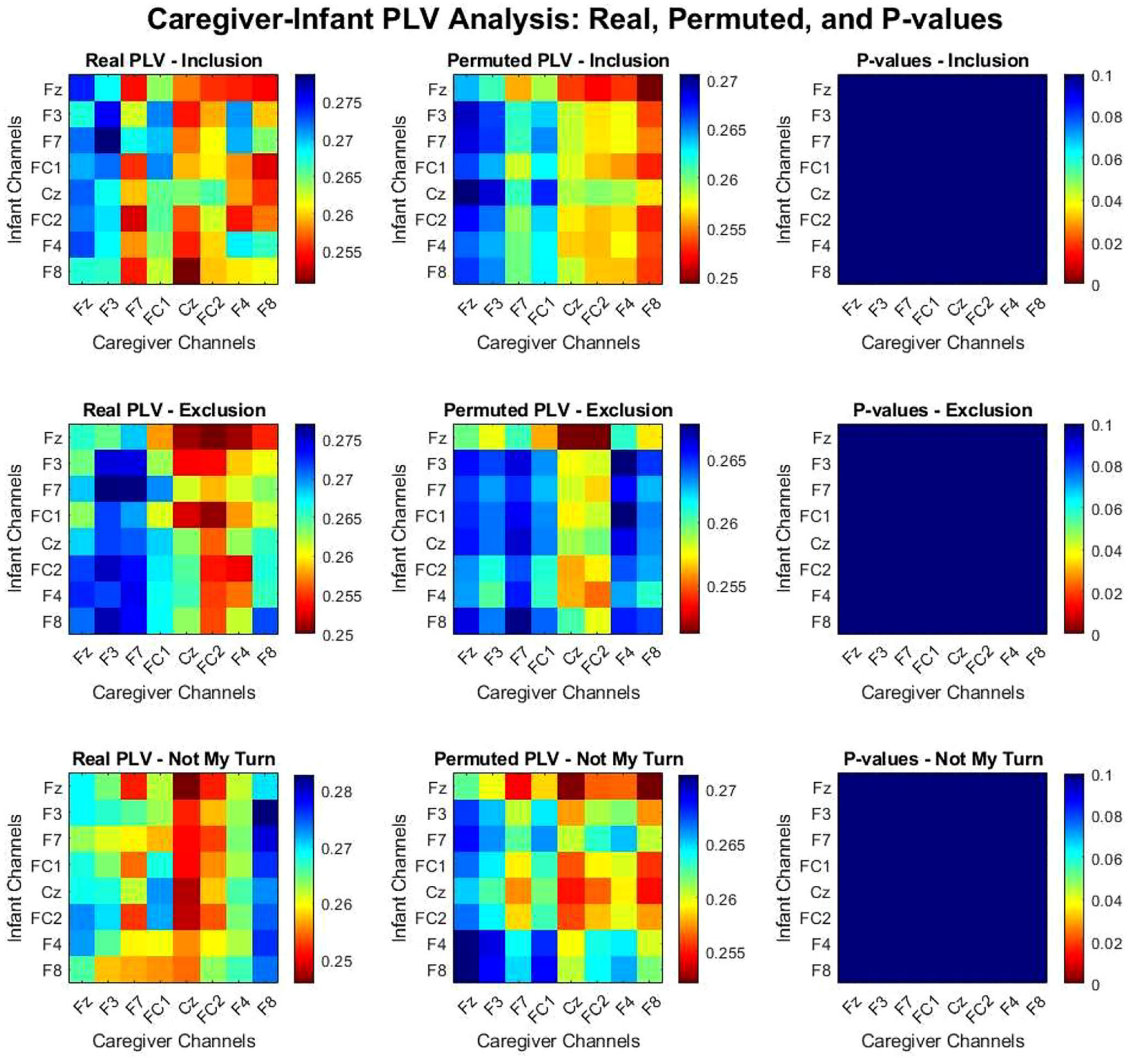
Results of PLV for real and permuted dyads and uncorrected *p*‐values. The figure shows the theta band brain‐to‐brain PLVs matrices of all 8 channels for real dyad (right matrixes) and surrogated dyad (middle matrixes) for each condition, and the corresponding uncorrected‐*p*‐value matrixes (left matrixes) for comparing real PLV to surrogated PLV. The resulting p‐maps were thresholded at *p *< .05.

We adopted a non‐parametric permutation test and paired samples t‐tests to investigate whether the observed PLV is higher compared to the PLV in permuted dyads and infant, caregiver and video control conditions, respectively. We first tested whether PLV significantly exceeded permuted PLV in inclusion, exclusion and not‐my‐turn trials. The results showed that observed PLV values did not significantly exceed permuted PLV (p_inclusion_ > .05, p_exclusion_ > .05, p_not my turn_ > .05, FDR‐corrected) in any of the trials (Figures 5 & 6). Moreover, we used paired samples t‐tests to compare the observed PLV with PLV in the control conditions. The result did not support higher observed PLV compared to all the control conditions, except in the video‐control condition for not my turn trial (Supplementary Materials 7). The results of power correlations and PDC are provided in Supplementary Material 8.

#### 3.6.2 Parental Bonding and PLV

The final sample size for the correlation between parental bonding and PLV comprised 34 infant–caregiver dyads.

To test the relation of parental bonding score and PLV between caregiver‐infant dyads, we ran a Spearman correlation analysis as the normal distribution was violated. The result showed a significant correlation in the not my turn trial (r_s_(32) = ‐0.30, *p* = 0.038), but there was no significant correlation in inclusion (r_s_(32) = ‐0.15, *p* = 0.189) and exclusion trials (r_s_(32) = ‐0.05, *p* = 0.374) (Supplementary Materials 9).

### 3.7 Exploratory Analysis

#### 3.7.1 Left‐lateralized Theta Power

As several studies have highlighted the importance of the left/medial frontal regions in theta power responses to ostracism (Crowley et al. 2010; Van Noordt et al. 2015a), we selected a cluster of left/medial electrode (F7, F3, Fz, and FC1). Theta power was extracted across these electrodes, and t‐tests were conducted to compare “exclusion” events with “inclusion” and “not my turn” events in both infants and caregivers. Infants showed higher theta power in “exclusion” compared to “not my turn” events (M_not my turn_ = 8.53, SD _not my turn_ = 1.05, M_exclusion_ = 9.01, SD_exclusion_ = 0.96), (*t* (33) = ‐1.95, *p* = 0.029, d = 0.33, BF = 28.55) and not in comparison to “inclusion” events (M_inclusion_ = 8.72, SD _inclusion_ = 0.88, M_exclusion_ = 9.01, SD_exclusion_ = 0.96), (*t* (33) = ‐1.40, *p* = 0.085, d = 0.24, BF = 9.82). However, caregivers did not show any significant differences between “exclusion” and “not my turn” events (M_not my turn_ = 6.12, SD _not my turn_ = 1.00, M_exclusion_ = 5.91, SD_exclusion_ = 0.93), (t (36) = 0.98, p = 0.833, d = ‐1.16, BF = 0.21) and nor “inclusion” events (M_inclusion_ = 6.04, SD _inclusion_ = 0.77, M_exclusion_ = 5.91, SD_exclusion_ = 0.93), (t (36) = 0.77, p = 0.779, d = ‐0.12, BF = 0.29).

#### 3.7.2 Correlation Between Affective and Behavioural Responses and Neural Markers

In order to explore which affective and behavioural responses contribute most significantly to the observed neural activity, 10 categories of correlation analyses were carried out using Pearson correlations. When the assumptions were violated, Spearman correlations were computed instead. P‐values were corrected using FDR: (1) correlation between caregivers’ affective and behavioural responses and theta power; (2) correlation between infants’ affective and behavioural responses and theta power; (3) cross‐correlation between caregivers’ affective and behavioural responses and infants’ theta power; (4) cross‐correlation between infants’ affective and behavioural responses and caregivers’ theta power; (5) correlations between caregivers’ affective and behavioural responses and alpha power; (6) correlations between infants’ affective and behavioural responses and alpha power; (7) cross‐correlations between caregivers’ affective and behavioural responses and infants’ alpha power; (8) cross‐correlations between infants’ affective and behavioural responses and caregivers’ alpha power; (9) correlation between PLV and caregivers’ affective and behavioural responses; (10) correlation between PLV and infants’ affective and behavioural responses.

The results showed no significant correlations between infants’ and caregivers’ theta and alpha power and their respective affective and behavioural responses (Tables 2, 3, 8 and 9 in Supplementary Materials 12). In terms of cross‐correlation analyses, no significant correlations were also found between infants’ alpha or theta power and caregivers’ emotional and behavioural responses, nor between caregivers’ alpha and theta power and infants’ emotional and behavioural responses. We only found a significant correlation between infants’ attention‐seeking behaviours and PLV during exclusion (r_s_(35) = 0.25, P_FDR_ = 0.047) (Table 7 in Supplementary Materials 12).

## 4 Discussion

The aim of the current study was to investigate the effect of ostracism on theta power, inter‐brain synchrony and affective and behavioural responses in infant‐caregiver dyads using the ball‐tossing game paradigm. To our knowledge, the current study is the first study to investigate infants’ responses to cues of ostracism experience at such an early age and to map how caregivers and infants synchronize at both neutral and behavioural levels in response to ostracism. Results showed that infants’ (but not caregivers’) theta power is higher in the exclusion block. Moreover, although both infants and caregivers showed affective and behavioural responses to ostracism, neural synchrony was not generally higher in ostracism compared to control conditions and it was highly dependent on the context. This may reflect a flexible modulation of neural synchrony in line with emotional co‐regulation processes.

### 4.1 Theta Power and Alpha Power

In accordance with our hypotheses, infants did show higher theta power in exclusion compared to not my turn and inclusion trials, and also higher alpha power in exclusion compared to not my turn trials. This is in line with previous research in populations of older children. For example, Tang et al. (2019) and Van Noordt et al. (2015b) also found that children showed heightened theta power in response to ostracism. While previous research has shown that thirteen‐ to fourteen‐month‐old infants exhibit behavioural responses to ostracism (Quadrelli et al. 2025), our findings are the first to demonstrate that even younger infants show early neural responses to ostracism, particularly in left/medial frontal regions.

Interestingly, in contrast to our hypothesis, there were no differences in alpha and theta power for caregivers in exclusion trials. Previous research findings were mixed: Cristofori et al. (2013) showed that adults showed higher theta power during exclusion, whereas Tang et al. (2019) observed higher theta power in response to not my turn events compared to exclusion events. At the behavioural level, Svetieva et al. (2019) also found that adults exhibit Duchenne smiling during exclusion, reflecting up‐regulation of positive emotion. Therefore, our findings confirm age‐related differences in neural responses to ostracism and further suggest that the presence of infants may activate caregivers’ regulatory responses, as discussed below.

To investigate which behavioural and affective responses map onto theta and alpha power, correlation analyses between neural responses and affective and behavioural responses were conducted. No significant results were found. This may suggest that alpha and theta power reflect a wide range of cognitive processes including a holistic cognitive evaluation of exclusion or monitoring and adjusting behaviours in response to conflict and implicit self‐control (Adamczyk and Wyczesany 2023; Cohen 2011; Cohen and Donner 2013), as all these processes can be evoked during exclusion rather than reflecting explicit affective responses in participants.

### 4.2 Inter‐brain Synchrony

Although at the individual level, findings showed that ostracism increased infants’ theta power, our hypothesis that ostracism would enhance inter‐brain synchrony between infants and caregivers was not confirmed. We found no significant differences in neural synchrony during ostracism compared to control and permuted conditions. However, PLV was higher in the main condition compared to caregiver and video‐control conditions only during not my turn trials. In contrast, behavioural analyses indicated that caregivers were sensitive to their infants’ ostracism, as reflected in synchrony in emotional expressions, social referencing, and visual attention. This dissociation between behavioural and neural synchrony may indicate a co‐regulation process—how caregivers adjust their internal states to support infants’ emotional balance and emerging self‐regulation (Feldman 2003; Butler and Randall 2012). Consistent with this, caregivers may downregulate negative emotions to buffer infants’ distress, which could reduce neural synchrony despite behavioural alignment. Prior work also suggests that synchrony may vary across levels (e.g., neural vs. physiological) and that flexible shifts between synchronized and desynchronized states may be adaptive (Wass et al., 2019). Importantly, this study is the first to examine infant–caregiver neural synchrony during ostracism, and further research is needed to replicate and clarify these findings.

Regarding the association between parental bonding and neural synchrony, the results showed that neural synchrony can be influenced by traits that dyads can bring to their interactions, with parental bonding being particularly influential in the “not my turn” trials. Although there was no significant association between PLV and parental bonding in exclusion and inclusion conditions, they showed the same trend, suggesting that when the parental bonding is better, the neural synchrony is higher (Ibáñez et al., 2017; Nguyen et al. 2020).

### 4.3 Affective and Behavioural Outcomes

As expected, infants showed more negative emotionality in the exclusion compared to the inclusion conditions, showing that infants as early as 4 months of age are sensitive to cues of ostracism experience and show negative emotionality during ostracism. Although alternative explanations for these findings could be proposed, they appear unlikely. For example, higher negative emotionality in ostracized infants could reflect boredom (Quadrelli et al. 2025). However, our results on attention‐seeking behaviours, supported by Bayes factors, show a trend for increased attention‐seeking during exclusion, suggesting infants’ responses reflect social sensitivity to ostracism rather than mere boredom. In terms of visual attention, infants showed higher attention during inclusion than exclusion, contrary to Quadrelli et al. (2025). Humans respond differently to ostracism depending on perceived opportunities for reaffiliation (Lyyra et al. 2017). In Quadrelli et al. (2025), experimenters smiled during exclusion, which may have signalled reengagement and increased infants’ attention. In contrast, experimenters in our study maintained neutral facial expressions. Consistent with this, infants showed increased attention during reinclusion, reflecting attempts to reconnect when reaffiliation is possible. Infants also did not exhibit increased social referencing during the exclusion. This null effect may be related to the study setup, as infants were seated on their caregivers’ laps, which may have limited their physical ability to look back toward the caregiver. This result for social referencing persists despite our efforts to code infants’ attempts to look toward the caregiver. Furthermore, the reinclusion phase partially recovered affective responses, with lower negative emotionality and reduced attention‐seeking compared to exclusion. These findings align with previous research in school‐aged children and adults (Tang and Richardson 2013; Zheng et al. 2024).

In line with our hypotheses, we found that caregivers showed less positive affect and more negative affect in the exclusion condition, supporting sharing of emotional states between caregivers and infants (Bowlby 1958). Moreover, caregivers showed more social referencing in the exclusion condition, showing that caregivers refer to their infants in ambiguous situations to respond to them accordingly and regulate their emotions. In terms of visual attention, caregivers, similar to infants, showed more visual attention in the inclusion condition, possibly because they did not receive any opportunity to reconnect and therefore withdrew from the interaction. However, they try to reconnect with others as soon as they see some affiliation cues in the reinclusion condition. Moreover, attention‐seeking behaviours suggest the same pattern; caregivers did not show higher attention‐seeking behaviour during the exclusion than the inclusion conditions, so they might withdraw from the interaction. Furthermore, the reinclusion phase appeared to help caregivers recover from negative emotional states and showed reduced social referencing compared to the exclusion condition. However, reinclusion did not restore positive emotionality or visual attention, nor caregivers’ positive emotionality. Previous research suggests that affective recovery occurs only when reinclusion matches or exceeds pre‐exclusion levels and may depend on individual differences such as self‐esteem (Kuang et al. 2024).

This study was the first to examine behavioural and neural responses to ostracism in 5‐ to 7‐month‐old infants and their caregivers. Combining EEG hyperscanning and behavioural measures allowed us to distinguish explicit and implicit synchrony during negative social experiences. Moreover, control conditions helped ensure that synchrony reflected shared emotional experience rather than joint movement. Some caveats should be considered in the interpretation of our findings. First, due to the live nature of the ball‐tossing game, caregivers supported infants differently during the ball game, which may have influenced infants’ sense of agency and experience of exclusion. In other words, there have been variabilities in how engaged caregivers were in the ball game compared to infants due to the naturalistic nature of the task, which may lead to variability in how intensely such effects are evoked and less neural modulation in response to exclusion among caregivers. Second, although during the debriefing, most of the caregivers reported that they understood that they were excluded during the game, we did not explicitly check the manipulation using a questionnaire. Additionally, not all dyads completed all control conditions due to time constraints. Furthermore, lack of longitudinal data limits our interpretation regarding developmental trajectories. Neural and behavioural responses to ostracism may become more pronounced or become more differentiated later in development. The present study is the first to demonstrate sensitivity to ostracism in infants aged 5 to 7 months, as well as caregiver‐infant coregulation during ostracism, and paves the way for future research to replicate these findings and longitudinally investigate the developmental trajectories. Particularly, we cannot directly conclude that infants can generate subjective feelings or evaluation of ostracism similarly to adults. Instead, our findings provide evidence of behavioural and neural sensitivity to cues associated with ostracism—a longitudinal study could provide further evidence. Given that overt emotional and behavioural responses did not directly map into neural responses during ostracism, neural responses should be interpreted cautiously, and it may show early cognitive evaluation of the situation, which can be a promising direction for future studies. Finally, the medial frontal cluster in our study was based on a 32‐electrode EEG montage, which included a relatively small subset of electrodes compared with previous studies. Therefore, future studies using a higher‐density EEG montage could further improve spatial specificity.

In summary, the present study is the first to demonstrate that infants as young as 5 months are sensitive to cues of ostracism at both neural and behavioural levels. This early sensitivity highlights the importance of emerging regulatory abilities and underscores why infants should not be excluded from social interactions, as such experiences may impact the development of social attention, emotional understanding, and later social, emotional, and cognitive skills. The current findings provide important insights into the early development of infants’ social cognition and into patterns of caregiver–infant coregulation during ostracism.

## Author Contributions

**Niloofar Goharbakhsh**: conceptualization, investigation, writing – original draft, writing – review and editing, methodology, validation, software, fomral analysis, project administration, data curation, visualization. **Louisa Kulke**: conceptualization, writing – review and editing, methodology, software, supervision, resources, funding acquisition, project administration.

## Funding

The authors have nothing to report.

## Ethics Statement

The study was reviewed and approved by the ethics board of University of Bremen (reference number 2024‐11).

## Conflicts of Interest

The authors declare no conflict of interest.

## Supporting information




**Supporting information**: desc70269‐supp‐0001‐SuppMat.docx

## Data Availability

The anonymised data are publicly accessible [https://osf.io/c3n6g/files/osfstorage]. The materials are presented in the manuscript (see methods section). There is a preregistration for all methods and analyses of the study [https://osf.io/dzh9s/overview].
