# Supplementary material for: Can You Feel My Pain? Neural‐Behavioural Changes in Caregiver–Infant Dyads During Ostracism
Source: Dev Sci. 2026 Aug 23;29(5):e70269. doi: 10.1111/desc.70269 (PMC13500035; doi:10.1111/desc.70269)
Supplement: Supplementary file 1 [file DESC-29-e70269-s001.docx]

# Supplementary Materials

## Supplementary Materials 1

First, we performed a power analysis for the within participants effect of the exclusion condition (inclusion vs exclusion) using the pwr.t.test function from the ‘pwr v1.3-0’ package. As our hypotheses are based on the study by Van Noordt et al. (2015b), we extracted their effect sizes (Cohen’s d), which were 1.08 for effects on peak theta in the exclusion condition compared to inclusion. Nine caregiver-infant dyads should be tested in total, with an alpha error of 0.05 and a power of 0.80. Secondly, we considered the study of Kayhan et al. (2022) to measure sample size for the neural synchronization among dyads. R was extracted, which is 0.42 for positive association between infant’s theta power and infant-mother’s theta power during a free play. To determine the required sample size for the correlation analysis, the wp.correlation function from the ‘WebPower v0.9-4’ package was employed. Thirty-three caregiver-infant dyads should be tested, with an alpha error of 0.05 and a power of 0.80. To be conservative, assuming the true effect size might be lower, we considered 33 participants (expected effect size of 0.42 with 0.80 power). To allow for full counterbalancing of the order of the confederates’ side, 34 participants need to be tested in total.

## Supplementary Materials 2

Affective and behavioural responses were coded on five scales (adapted from Quadrelli et al., 2025) for infants as follows: (1) positive emotionality as a sum of the smile and happy vocalization, (2) negative emotionality as a sum of the angry and sad expressions, angry vocalization, withdrawal from the game, distracting themselves from the game/looking at other side and seeking support, (3) visual attention as a sum of looking at other players and looking at the ball/hand, (4) attention-seeking behavior as a sum of the body posture toward other players, looking at other players, and moving hand and arms to catch players’ attention, and (5) social referencing as a sum of looking back at caregivers or trying to look back at caregivers. For caregivers, positive and negative emotionality were coded on similar subscales, except that happy and angry vocalization were excluded as adults did not openly show these vocalisations. For attention-seeking behaviours, we also added one more subscale: talking to player and infant. Social referencing was coded as a sum of looking at infants or trying to look at infants.

The inter-rater reliability (Fleiss' Kappa) was computed for around 10 % of the videos (caregivers: positive emotionality K < 0.90, negative emotionality k = 1, attention-seeking behaviour K < 0.86, visual attention K < 0.81, social referencing K < 0.84; infants: positive emotionality K < 0.73, negative emotionality k < 0.82, attention-seeking behaviour K < 0.82, visual attention K < 0.81, social referencing K < 0.73).

## Supplementary Materials 3

The mean and SD of the removed ICA components and interpolated channels for the included dataset for theta and alpha power are as follows; infants: M_ICA_ = 0.26, SD _ICA_ = 0.51, M _interpolated_ = 0.05, SD _interpolated_ = 0.23, caregivers: M_ICA_ = 1.70, SD _ICA_ = 0.52, M _interpolated_ = 0.02, SD _interpolated_ = 0.16.

The mean and SD of the remaining trials after preprocessing for theta and alpha power analyses are as follows; infants: M_inclusion_ = 12.8, SD_inclusion_ = 1.39, M_exclusion_ = 14.2, SD_exclusion_ = 2.02 and M_not my turn_ = 12.8, SD_not my turn_ = 1.53, caregivers: M_inclusion_ = 13.3, SD_inclusion_ = 1.60, M_exclusion_ = 15.2, SD_exclusion_ = 1.67 and M_not my turn_ = 13.4, SD_not my turn_ = 1.34).

## Supplementary Materials 4

The mean and SD of the removed ICA components and interpolated channels for the included dataset for inter-brain synchrony analyses are as follows: infants: M_ICA_ = 0.26, SD _ICA_ = 0.51, M _interpolated_ = 0.05, SD _interpolated_ = 0.23, caregivers: M_ICA_ = 1.71, SD _ICA_ = 0.52, M _interpolated_ = 0.02, SD _interpolated_ = 0.17.

After preprocessing the mean and SD of the remaining trials for inter-brain synchrony are infants: M_inclusion_ = 12.08, SD_inclusion_ = 1.39, M_exclusion_ = 14.2, SD_exclusion_ = 2.02 and M_not my turn_ = 12.8, SD_not my turn_ = 1.53, caregivers: M_inclusion_ = 13.2, SD_inclusion_ = 1.65, M_exclusion_ = 15.1, SD_exclusion_ = 1.73 and M_not my turn_ = 13.3, SD_not my turn_ = 1.39).

## Supplementary Materials 5

**Power correlations**: we computed the power correlation for theta band activity using Spearman’s correlation between the matched trials of caregivers and infants. Spearman’s correlation was used as it is generally preferred due to its robustness to non-normally distributed data and less sensitivity to outliers (Cohen, 2014; Marriott Haresign et al., 2022). First, the *FiltHilb* function (Marriott Haresign et al., 2022) was used to narrow band filter the data. This was used in conjunction with the Hilbert transform to obtain the **analytic signal** of each frequency bin and compute the theta power. Subsequently, the *tfPow_corr* (Marriott Haresign et al., 2022) function was applied to compute power correlations for each trial. Trials were separated according to experimental conditions (inclusion, exclusion, and not my turn). We then randomly paired infants and caregivers from different dyads 1000 times, calculating the same Spearman correlation for each random pairing to create a null distribution, representing correlations expected by chance. P-values were calculated as the proportion of random correlations exceeding the observed correlation. This procedure allowed us to determine whether the observed correlation was significantly greater than expected by chance, providing a robust test against spurious correlations due to shared task in infant-caregiver dyads.

**Partial directed coherence (PDC):** Partial directed coherence (PDC) was also computed, which is based on the principles of Granger (Baccalá & Sameshima, 2001). It provides information about how strongly one time series can predict another across different frequencies (the equation can be found in Marriott Haresign et al., 2023). It is derived from the coefficients of a multivariate autoregressive (MVAR) model. PDC was computed in both directions (Infant → Caregiver and Caregiver → Infant) across the 3–6 Hz frequency band (Marriott Haresign et al., 2023) using a model order of 5. The **model order** specifies how many past time points of the signals are used to predict their current values. In line with previous literature (Leong et al., 2017; Marriott Haresign et al., 2023), we selected a model order of 5; Jansen et al. (1981) reported that a 5 model order was sufficient in 90% of cases to capture variance in EEG time series data, and Vaz et al. (1987) suggested that a model order of 5 adequately represents 1- or 2-s EEG segments, although higher orders may be needed when the signal lacks distinctive features. After computing PDC for the matched trials, they were separated according to experimental conditions (inclusion, exclusion, and not my turn). We then randomly paired infants and caregivers from different dyads 1000 times to make a null distribution, representing PDC expected by chance. P-values were calculated as the proportion of random PDC exceeding the observed PDC. This procedure allowed us to determine whether the observed PDC was significantly greater than expected by chance, providing a robust test against spurious correlations due to shared task in infant-caregivers dyads.

## Supplementary Materials 6

## Infant

We carried out two paired samples t-tests to compare alpha power during “exclusion” events with the “not my turn” and “inclusion” events. The result showed that alpha power was higher during “exclusion” events compared to the “not my turn” events (M_not my turn_ = 7.01, SD_not my turn_ = 1.06, M_exclusion_ = 7.46, SD_exclusion_ = 0.96), (t (33) = -2.10, p = 0.021, d = 0.36, BF = 38.60) but not compared to the “inclusion” events (M_inclusion_= 7.23, SD_inclusion_ = 1.34, M_exclusion_ = 7.46, SD_exclusion_ = 9.96), (t (33) = -0.91, p = 0.184, d = 0.15, BF = 4.18) (Figure 1).

## Caregivers

Two paired samples t-test was carried out to compare alpha power during “exclusion” events with the “not my turn” and “inclusion” events. There were also no significant differences in alpha power between “exclusion” and “not my turn” events (M_not my turn_ = 6.02, SD_not my turn_ = 1.03, M_exclusion_ = 5.59, SD_exclusion_ = 1.11), (t (36) = 2.48, p = 0.991, d = -0.40, BF = 0.01) and “exclusion” and “inclusion” events (M_inclusion_ = 5.83, SD_inclusion_ = 1.06, M_exclusion_ = 5.59, SD_exclusion_ = 1.11), (t (36) = 1.16, p = 0.874, d = -0.19, BF = 0.15). However, Bayes Factor supported anecdotal evidence for alternative hypothesis for alpha power differences between “exclusion” and “not my turn” events (Figure 1).

**Figure 1**. The alpha power in infants (purple background) (a) and caregivers (b) during inclusion, exclusion and not my turn trials

## Supplementary Materials 7

| **Control condition** | **Mean (SD)** | **t** | **df** | **P** | **d** | **BF** |
| --- | --- | --- | --- | --- | --- | --- |
| infant-control condition | M_inclusion_= 0.25 (0.02) | -0.21 | 9 | 0.583 | -0.07 | 0.73 |
|  | M_inclusion-control_=0.25 (0.02) |  |  |  |  |  |
|  | M_exclusion_= 0.25 (0.03) | -0.20 | 9 | 0.579 | -0.07 | 0.74 |
|  | M_exclusion_ _-control_ = 0.25 (0.02) |  |  |  |  |  |
|  | M_not my turn_= 0.26 (0.03) | 1.18 | 9 | 0.132 | -0.38 | 5.60 |
|  | M_notmyturn-control_ = 0.24 (0.01) |  |  |  |  |  |
| caregiver-control condition | M_inclusion_ = 0.26 (0.02) | -0.31 | 10 | 0.620 | -0.09 | 0.63 |
|  | M_inclusion-control_ = 0.26 (0.02) |  |  |  |  |  |
|  | M_exclusion_= 0.25 (0.02) | -1.30 | 10 | 0.889 | -0.39 | 0.14 |
|  | M_exclusion_ _-control_ = 0.26 (0.03) |  |  |  |  |  |
|  | M_not my turn_= 0.27 (0.02) | 1.54 | 10 | 0.076 | 0.47 | 9.81 |
|  | M_not my turn-control_ = 0.25 (0.02) |  |  |  |  |  |
| video-control condition | M_inclusion_ = 0.27 (0.02) | 0.56 | 16 | 0.288 | 0.14 | 2.34 |
|  | M_inclusion-control_ = 0.26 (0.03) |  |  |  |  |  |
|  | M_exclusion_= 0.25 (0.02) | -0.51 | 16 | 0.694 | -0.13 | 0.46 |
|  | M_exclusion_ _-control_ = 0.26 (0.03) |  |  |  |  |  |
|  | M_not my turn_ = 0.27 (0.02) | 2.07 | 16 | 0.027* | 0.50 | 27.84 |
|  | M_not my turn-control_ = 0.25 (0.02) |  |  |  |  |  |

**Table 1**. Results of one-tailed t tests for comparing PLV to control conditions including infant-control condition, caregiver-control condition and video-control condition. BF= Bayes factor.

## Supplementary Materials 8

### Power correlation

To test whether the observed power correlation is higher compared to the power correlation of permuted dyads, a non-parametric permutation test was carried out. We tested whether theta power correlation significantly exceeded permuted power correlation in inclusion, exclusion and not-my-turn trials. The results showed that observed theta power correlation did not significantly exceed permuted theta power correlation (p_inclusion_ > .05, p_exclusion_ > .05 p_not my turn_ > .05, FDR-corrected) (Figures 2 & 3) in any of the trials.

**Figure 2**. Results of permutation tests for theta power correlation analysis. Red dashed lines indicate averaged observed correlation in theta, black dashed lines indicate the threshold for p < 0.05.

**Figure 3**. Results of theta power correlation for real and permuted dyads and uncorrected p-values. The figure shows the theta power correlation matrices of all 8 channels for real dyad (first row) and permuted dyad (middle row) for each condition, and the corresponding uncorrected-p-value matrixes (last row) for comparing real power correlation to permuted correlation. The resulting p-maps were thresholded at  p < .05.

### Partial-directed coherence (PDC)

To investigate whether the observed PDC is higher compared to permuted PDC, a non-parametric permutation test was used. The result showed that the observed PDC did not significantly exceed permuted PDC (p_inclusion_ > .05, p_exclusion_ > .05, p_not my turn_ > .05, FDR-corrected) (Figure 4) in any of the trials and in both directions (Infant → Caregiver and Caregiver → Infant). However, 1 p-value in inclusion and 2 p-values in exclusion trials were significant out of 64 channel-by-channel comparisons, respectively (> .05, for uncorrected p-values) (Figure 5).

**Figure 4**. Results of permutation tests for PDC. Red dashed lines indicate averaged observed PDC in theta, black dashed lines indicate the threshold for p < 0.05.

**Figure 5**. Results of PDC for infant→caregivers and caregiver→infant and uncorrected p-values. The figure shows PDC matrices of all 8 channels for real dyad for infant→caregiver (first row) and for caregive→infant (middle row), and the corresponding uncorrected-p-value matrixes (third and fourth rows) for comparing real PDC to permuted PDC. The resulting p-maps were thresholded at  p < .05.

## Supplementary Materials 9

**Figure 6**. Results of the Spearman correlation between PLV and PBQ. PBQ= parental bonding scores, PLV = phase locking value.

## Supplementary Materials 10

### Affective and Behavioural outcomes in reinclusion condition

#### Infants

To test whether infants showed more positive emotionality and less negative emotionality and social referencing in reinclusion compared to exclusion conditions, one paired samples t-test and two Wilcoxon tests were performed. In addition, Bayes Factors were computed. The result confirmed no significant difference in positive emotionality (M_reinclusion_ = 14.04, SD_reinclusion_ = 18.27, M_exclusion_ = 13.82, SD_exclusion_ = 20.27), (W = 328, p = 0.364, r = 0.06, BF = 1.14) and in social referencing (M_reinclusion_ = 0.17, SD_reinclusion_ = 3.32, M_exclusion_ = 0.55, SD_exclusion_ = 1.61), (W = 24, p = 0.255, r = 0.11, BF = 0.46) between reinclusion and exclusion conditions. However, infants showed less negative emotionality in the reinclusion compared to exclusion conditions (M_reinclusion_ = 32.02, SD_reinclusion_ = 35.76, M_exclusion_ = 41.37, SD_exclusion_ = 34.38), (t (40) = 1.85, p = 0.035, d = 0.29, BF = 23.94). To test whether there is a difference in attention-seeking behaviour and visual attention in the reinclusion and exclusion conditions, we ran one paired samples t-test and one Wilcoxon test. The result showed no significant difference in attention-seeking behaviours (M_reinclusion_ = 10.68, SD_inclusion_ = 12.64, M_exclusion_ = 18.07, SD_exclusion_ = 20.59), (W = 407, p = 0.134, r = 0.22, BF = 1.17), however, Bayes Factor showed anecdotal evidence for the alternative hypothesis. In terms of visual attention, there was no significant difference (M_reinclusion_ = 76.05, SD_reinclusion_ = 20.23, M_exclusion_ = 75.49, SD_exclusion_ = 20.02), (t (40) = -0.17, p = 0.867, d = -0.03, BF = 0.17) in inclusion compared to exclusion conditions.

#### Caregivers

To test whether caregivers also showed more positive emotionality and less negative emotionality and social referencing in reinclusion compared to exclusion conditions, three paired samples t-tests were performed. In addition, Bayes Factors were computed. The result confirmed no significant difference in positive emotionality (M_reinclusion_ = 39.58, SD_reinclusion_ = 32.96, M_exclusion_ = 43.46, SD_exclusion_ = 34.42), (t (40) = 1.09, p = 0.860, d = 0.17, BF = 0.17) between reinclusion and exclusion conditions. However, caregivers showed less social referencing (M_reinclusion_ = 9.12, SD_reinclusion_ = 10.29, M_exclusion_ = 16.48, SD_exclusion_ = 15.88), (t (40) = 3.13, p = 0.003, d = 0.49, BF = 10.90) and less negative emotionality (M_reinclusion_ = 9/97, SD_reinclusion_ = 10.85, M_exclusion_ = 16.9, SD_exclusion_ = 16.29), (t (40) = 2.84, p = 0.003, d = 0.44, BF = 226.67) in reinclusion compared to exclusion conditions. To test whether there is a difference in attention-seeking behaviour and visual attention in reinclusion and exclusion conditions, we ran one Wilcoxon test and one paired samples t-test. The result showed no significant difference in attention-seeking behaviours (M_reinclusion_ = 8.59, SD_inclusion_ = 17.24, M_exclusion_ = 13.27, SD_exclusion_ = 29.69), (W = 152, p = 0.210, r = 0.14, BF = 0.62. In terms of visual attention, caregivers showed more visual attention in reinclusion condition compared to the exclusion condition (M_reinclusion_ = 86.29, SD_reinclusion_ = 11.65, M_exclusion_ = 73.68, SD_exclusion_ = 18.82), (t (40) = -4.67, p < 0.001, d = -0.73, BF = 660.81).

## Supplementary Materials 11

**Figure 7**. Results of Spearman correlation for affective and behavioural outcomes (exclusion block)

**Figure 8**. Results of Spearman correlation for affective and behavioural outcomes (inclusion block)

**Figure 9**. Results of Spearman correlation for affective and behavioural outcomes (reinclusion block)

## Supplementary Materials 12

### Correlation between theta power and emotional and behavioural responses

|  | Positive emotionality | Negative emotionality | Attention seeking behaviours | Visual attention | Social referencing |
| --- | --- | --- | --- | --- | --- |
| Theta power in inclusion | r_p_(35) = -0.01 P = 0.928 | r_s_(35) = 0.08  P = 0. 928 | r_s_(35) = 0.30  P = 0.338 | r_s_(35) = 0.15  P = 0.892 | r_s_(35) =0.04  P = 0.928 |
| Theta power in not my turn | r_p_(35) = -0.01  P = 0.951 | r_s_(35) = 0.03  P = 0.951 | r_s_(35) = 0.16  P = 0.951 | r_s_(35) = 0.05  P = 0.951 | r_s_(35) = 0.03  P = 0.951 |
| Theta power in exclusion | r_s_(35) = 0.04  P = 0.963 | r_s_(35) = 0.02  P = 0.963 | r_s_(35) = 0.15  P = 0.963 | r_p_(35) = -0.03  P = 0.963 | r_s_(35) = 0.008  P = 0.963 |

**Table 2.** Correlation analysis between caregivers’ theta power and affective and behavioural responses for each block. r_p_ = Pearson corelation; r_s_ = Spearman corelation. All p-values are FDR-corrected.

|  | Positive emotionality | Negative emotionality | Attention seeking behaviours | Visual attention | Social referencing |
| --- | --- | --- | --- | --- | --- |
| Theta power in inclusion | r_s_(32) = -0.17 P = 0.526 | r_s_(32) = 0.08  P = 0.812 | r_s_(32) = -0.006  P = 0.973 | r_s_(32) = -0.18  P = 0.526 | r_s_(32) =0.20  P = 0.526 |
| Theta power in not my turn | r_s_(32) = 0.25  P = 0.318 | r_s_(32) = -0.28  P = 0.318 | r_s_(32) = -0.23  P = 0.318 | r_s_(32) = 0.06  P = 0.780 | r_s_(32) = -0.05  P = 0.780 |
| Theta power in exclusion | r_s_(32) = -0.20  P = 0.514 | r_s_(32) = -0.14  P = 0.514 | r_s_(32) = -0.23  P = 0.514 | r_s_(32) = 0.16  P = 0.514 | r_s_(32) = -0.11  P = 0.533 |

**Table 3.** Correlation analysis between infants’ theta power and affective and behavioural responses for each block. r_p_ = Pearson corelation; r_s_ = Spearman corelation. All p-values are FDR-corrected.

|  | Positive emotionality | Negative emotionality | Attention seeking behaviours | Visual attention | Social referencing |
| --- | --- | --- | --- | --- | --- |
| Theta power in inclusion | r_p_(32) = -0.37 P = 0.154 | r_s_(32) = -0.12  P = 0.597 | r_s_(32) = 0.06  P = 0.713 | r_s_(32) = 0.20  P = 0.408 | r_s_(32) =-0.21  P = 0.408 |
| Theta power in not my turn | r_p_(32) = -0.33  P = 0.148 | r_s_(32) = -0.27  P = 0.204 | r_s_(32) = 0.11  P = 0.504 | r_s_(32) = 0.23  P = 0.237 | r_s_(32) = -0.32  P = 0.148 |
| Theta power in exclusion | r_s_(32) = -0.02  P = 0.889 | r_s_(32) = -0.05  P = 0.899 | r_s_(32) = -0.06  P = 0.899 | r_p_ (32) = 0.31  P = 0.363 | r_s_ (32) = -0.04  P = 0.889 |

**Table 4.** Cross correlation analysis between infants’ theta power and caregivers’ affective and behavioural responses for each block. r_p_ = Pearson corelation; r_s_ = Spearman corelation. All p-values are FDR-corrected.

|  | Positive emotionality | Negative emotionality | Attention seeking behaviours | Visual attention | Social referencing |
| --- | --- | --- | --- | --- | --- |
| Theta power in inclusion | r_s_(35) = 0.27  P = 0.466 | r_s_(35) = -0.10  P = 0.762 | r_s_(35) = 0.03  P = 0.846 | r_s_(35) = -0.22  P = 0.466 | r_s_(35) =0.08  P = 0.762 |
| Theta power in not my turn | r_s_(35) = -0.21  P = 0.724 | r_s_(35) = -0.09  P = 0.873 | r_s_(35) = -0.06  P = 0.873 | r_s_(35) = -0.008  P = 0.964 | r_s_(35) = -0.17  P = 0.724 |
| Theta power in exclusion | r_s_(35) = 0.07  P = 0.733 | r_s_(35) = -0.05  P = 0.733 | r_s_(35) = -0.09  P = 0.733 | r_s_(35) = 0.19  P = 0.733 | r_s_(35) = 0.08  P = 0.733 |

**Table 5.** Cross correlation analysis between caregivers’ theta power and infants’ affective and behavioural responses for each block. r_p_ = Pearson corelation; r_s_ = Spearman corelation. All p-values are FDR-corrected.

### Correlation between PLV and emotional and behavioural responses

|  | Positive emotionality | Negative emotionality | Attention seeking behaviours | Visual attention | Social referencing |
| --- | --- | --- | --- | --- | --- |
| PLV in inclusion | r_s_(35) = -0.08  P = 0.586 | r_s_(35) = -0.07  P = 0.586 | r_s_(35) = -0.03  P = 0.707 | r_s_(35) = 0.16  P = 0.516 | r_s_(35) =-0.09  P = 0.586 |
| PLV in not my turn | r_s_(35) = -0.07  P = 0.584 | r_s_(35) = -0.10  P = 0.478 | r_s_(35) = -0.15  P = 0.478 | r_s_(35) = -0.02  P = 0.788 | r_s_(35) = -0.11  P = 0.478 |
| PLV in exclusion | r_s_(35) = 0.16  P = 0.419 | r_s_(35) = -0.11  P = 0.419 | r_s_(35) = 0.06  P = 0.582 | r_s_(35) = 0.05  P = 0.582 | r_s_(35) = -0.11  P = 0.419 |

**Table 6.** Correlation analysis between PLV and caregivers’ affective and behavioural responses for each block. r_p_ = Pearson corelation; r_s_ = Spearman corelation. All p-values are FDR-corrected.

|  | Positive emotionality | Negative emotionality | Attention seeking behaviours | Visual attention | Social referencing |
| --- | --- | --- | --- | --- | --- |
| PLV in inclusion | r_s_(35) = -0.02  P = 0.933 | r_s_(35) = -0.22  P = 0.111 | r_s_(35) = 0.17  P = 0.215 | r_s_(35) = 0.008  P = 0.933 | r_s_(35) =0.01  P = 0.933 |
| PLV in not my turn | r_s_(35) = -0.05  P = 0.598 | r_s_(35) = -0.08  P = 0.470 | r_s_(35) = 0.22  P = 0.104 | r_s_(35) = 0.20  P = 0.104 | r_s_(35) = -0.16  P = 0.179 |
| PLV in exclusion | r_s_(35) = -0.16  P = 0.176 | r_s_(35) = -0.08  P = 0.518 | r_s_(35) = 0.25  P = 0.047* | r_s_(35) = 0.16  P = 0.176 | r_s_(35) = -0.01  P = 0.890 |

**Table 7.** Correlation analysis between PLV and infants’ affective and behavioural responses for each block. r_p_ = Pearson corelation; r_s_ = Spearman corelation. All p-values are FDR-corrected.

### Correlation between alpha power and emotional and behavioural responses

|  | Positive emotionality | Negative emotionality | Attention seeking behaviours | Visual attention | Social referencing |
| --- | --- | --- | --- | --- | --- |
| Alpha power in inclusion | r_p_(35) = -0.18  P = 0.947 | r_s_(35) = -0.01  P = 0.947 | r_s_(35) = -0.11  P = 0.947 | r_s_(35) = -0.06  P = 0.947 | r_s_(35) =0.02  P = 0.947 |
| Alpha power in not my turn | r_p_(35) = 0.18  P = 0.710 | r_s_(35) = 0.10  P = 0.775 | r_s_(35) = 0.27  P = 0.511 | r_s_(35) = -0.04  P = 0.799 | r_s_(35) = 0.08  P = 0.775 |
| Alpha power in exclusion | r_s_(35) = 0.03  P = 0.822 | r_s_(35) = 0.05  P = 0.822 | r_s_(35) = 0.33  P = 0.211 | r_p_(35) = 0.08  P = 0.822 | r_s_(35) = 0.05  P = 0.822 |

**Table 8.** Correlation analysis between caregivers’ alpha power and affective and behavioural responses for each block. r_p_ = Pearson corelation; r_s_ = Spearman corelation. All p-values are FDR-corrected.

|  | Positive emotionality | Negative emotionality | Attention seeking behaviours | Visual attention | Social referencing |
| --- | --- | --- | --- | --- | --- |
| Alpha power in inclusion | r_s_(32) = 0.08 P = 0.778 | r_s_(32) = -0.04  P = 0.804 | r_s_(32) = 0.16  P = 0.678 | r_s_(32) = -0.29  P = 0.484 | r_s_(32) =0.14  P = 0.678 |
| Alpha power in not my turn | r_s_(32) = -0.08  P = 0.921 | r_s_(32) = -0.16  P = 0.885 | r_s_(32) = -0.23  P = 0.885 | r_s_(32) = -0.06  P = 0.921 | r_s_(32) = -0.01  P = 0.929 |
| Alpha power in exclusion | r_s_(32) = -0.13  P = 0.675 | r_s_(32) = -0.10  P = 0.675 | r_s_(32) = -0.10  P = 0.675 | r_s_(32) = 0.12  P = 0.675 | r_s_(32) = -0.07  P = 0.675 |

**Table 9.** Correlation analysis between infants’ alpha power and affective and behavioural responses for each block. r_p_ = Pearson corelation; r_s_ = Spearman corelation. All p-values are FDR-corrected.

|  | Positive emotionality | Negative emotionality | Attention seeking behaviours | Visual attention | Social referencing |
| --- | --- | --- | --- | --- | --- |
| Alpha power in inclusion | r_p_(32) = -0.33 P = 0.285 | r_s_(32) = -0.007  P = 0.996 | r_s_(32) = 0.09  P = 0.966 | r_s_(32) = 0.14  P = 0.966 | r_s_(32) =-0.01  P = 0.966 |
| Alpha power in not my turn | r_p_(32) = -0.39  P = 0.107 | r_s_(32) = -0.007  P = 0.968 | r_s_(32) = 0.14  P = 0. 968 | r_s_(32) = -00.8  P = 0. 968 | r_s_(32) = -0.05  P = 0. 968 |
| Alpha power in exclusion | r_s_(32) = 0.05  P = 0.779 | r_s_(32) = -0.12  P = 0.779 | r_s_(32) = -0.05  P = 0.779 | r_p_ (32) = 0.29  P = 0.473 | r_s_ (32) = -0.10  P = 0.779 |

**Table 10.** Cross correlation analysis between infants’ alpha power and caregivers’ affective and behavioural responses for each block. r_p_ = Pearson corelation; r_s_ = Spearman corelation. All p-values are FDR-corrected.

|  | Positive emotionality | Negative emotionality | Attention seeking behaviours | Visual attention | Social referencing |
| --- | --- | --- | --- | --- | --- |
| Alpha power in inclusion | r_s_(35) = -0.26  P = 0.289 | r_s_(35) = 0.01  P = 0.955 | r_s_(35) = -0.31  P = 0.270 | r_s_(35) = 0.07  P = 0.828 | r_s_(35) =-0.14  P = 0.671 |
| Alpha power in not my turn | r_s_(35) = -0.12  P = 0.533 | r_s_(35) = -0.12  P = 0. 533 | r_s_(35) = 0.15  P = 0. 533 | r_s_(35) = -0.10  P = 0533 | r_s_(35) = -0.23  P = 0. 533 |
| Alpha power in exclusion | r_s_(35) = 0.04  P = 0.837 | r_s_(35) = 0.03  P = 0.837 | r_s_(35) = -0.22  P = 0.574 | r_s_(35) = -0.20  P = 0.574 | r_s_(35) = -0.13  P = 0.738 |

**Table 11.** Cross correlation analysis between caregivers’ alpha power and infants’ affective and behavioural responses for each block. r_p_ = Pearson corelation; r_s_ = Spearman corelation. All p-values are FDR-corrected.
